# Combined Repair of Coarctation of the Aorta and Long-Segment Tracheal Stenosis in a Neonate

**DOI:** 10.1016/j.atssr.2023.06.015

**Published:** 2023-07-28

**Authors:** Madonna E. Lee, Sarah E. Maurrasse, Heidi Boules, Erin Faherty, Michael Weinstock, Michael G. Caty

**Affiliations:** 1 Department of Cardiac Surgery, Yale School of Medicine, New Haven, Connecticut; 2 Section of Pediatric Otolaryngology, Yale School of Medicine, New Haven, Connecticut; 3 Division of Pediatric Anesthesia, Yale School of Medicine, New Haven, Connecticut; 4 Section of Pediatric Cardiology, Yale School of Medicine, New Haven, Connecticut; 5 Division of Pediatric Surgery, Yale School of Medicine, New Haven, Connecticut

## Abstract

Complete tracheal rings requiring concomitant cardiac and airway repair are typically described in association with pulmonary artery sling. We report a newborn case of coarctation of the aorta and incidental discovery of asymptomatic severe tracheal stenosis at the time of attempted coarctation repair. This case illustrates the importance of a thorough airway evaluation, multidisciplinary coordination of pediatric surgical and anesthesia specialties, and detailed preoperative planning to ensure successful surgical treatment of this rare entity of combined neonatal congenital cardiac and airway disease.

Concomitant tracheal rings and congenital heart disease are classically described in infants with pulmonary artery sling.^1^ Most infants undergo airway reconstruction for severe tracheal stenosis between 4 and 8 months.^2^, ^3^, ^4^ Although rare, a spectrum of congenital heart disease can occur with long-segment tracheal stenosis.^2^^,^^5^ The incidence of congenital cardiac disease excluding pulmonary artery sling and severe tracheal stenosis in neonates is unknown. Our neonate presented with coarctation of the aorta (CoA) and an incidental finding of severe tracheal stenosis due to an inability to be intubated.

A 2.9-kg girl was delivered with prenatal concern for CoA. Shortly after birth, an upper/lower blood pressure differential developed with systemic hypoperfusion. Prostaglandin infusion was initiated. Transthoracic echocardiography confirmed severe juxtaductal CoA with a mildly hypoplastic aortic arch (*z* score, −2) and bicuspid aortic valve with no stenosis or regurgitation. The patient was breathing comfortably on room air. Repair of CoA was planned through left thoracotomy; however, in the operating room, intubation was challenging because of inability to pass a 3.5-mm or subsequently a 3.0-mm endotracheal tube (ETT), despite adequate view of the vocal cords. A pediatric otolaryngologist was consulted, and an uncuffed 2.5-mm ETT fit tightly into the airway. Flexible bronchoscopy identified a discrete tracheal narrowing just below the vocal cords. The case was discontinued, and computed tomography angiography of the neck and chest was performed. A mildly hypoplastic aortic arch, CoA, and bovine arch were identified, with no evidence of vascular rings (Figure 1A). At the thoracic inlet, there was tracheal narrowing (Figure 1B). Bronchoscopic evaluation revealed severe tracheal stenosis beginning in the cervical trachea due to complete tracheal rings extending distally to the carina (Figure 2A). A 2.5-mm bronchoscope was unable to be passed into the stenotic segment.

**Figure 1:**
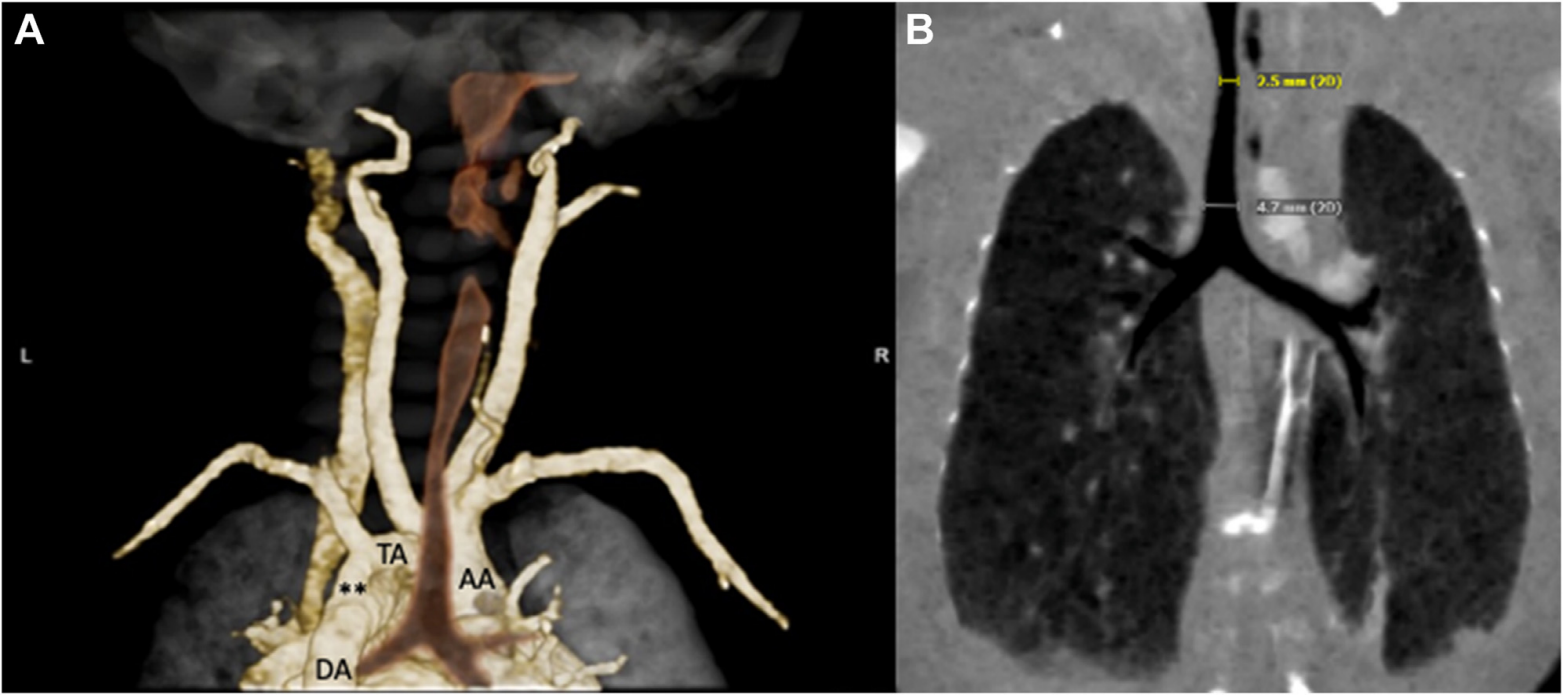
Neck and chest computed tomography angiography, coronal views. (A) Hypoplastic transverse aortic arch (TA) and coarctation (∗∗) with bovine arch. (B) Thoracic tracheal narrowing. (AA, ascending aorta; DA, descending aorta.)

**Figure 2:**
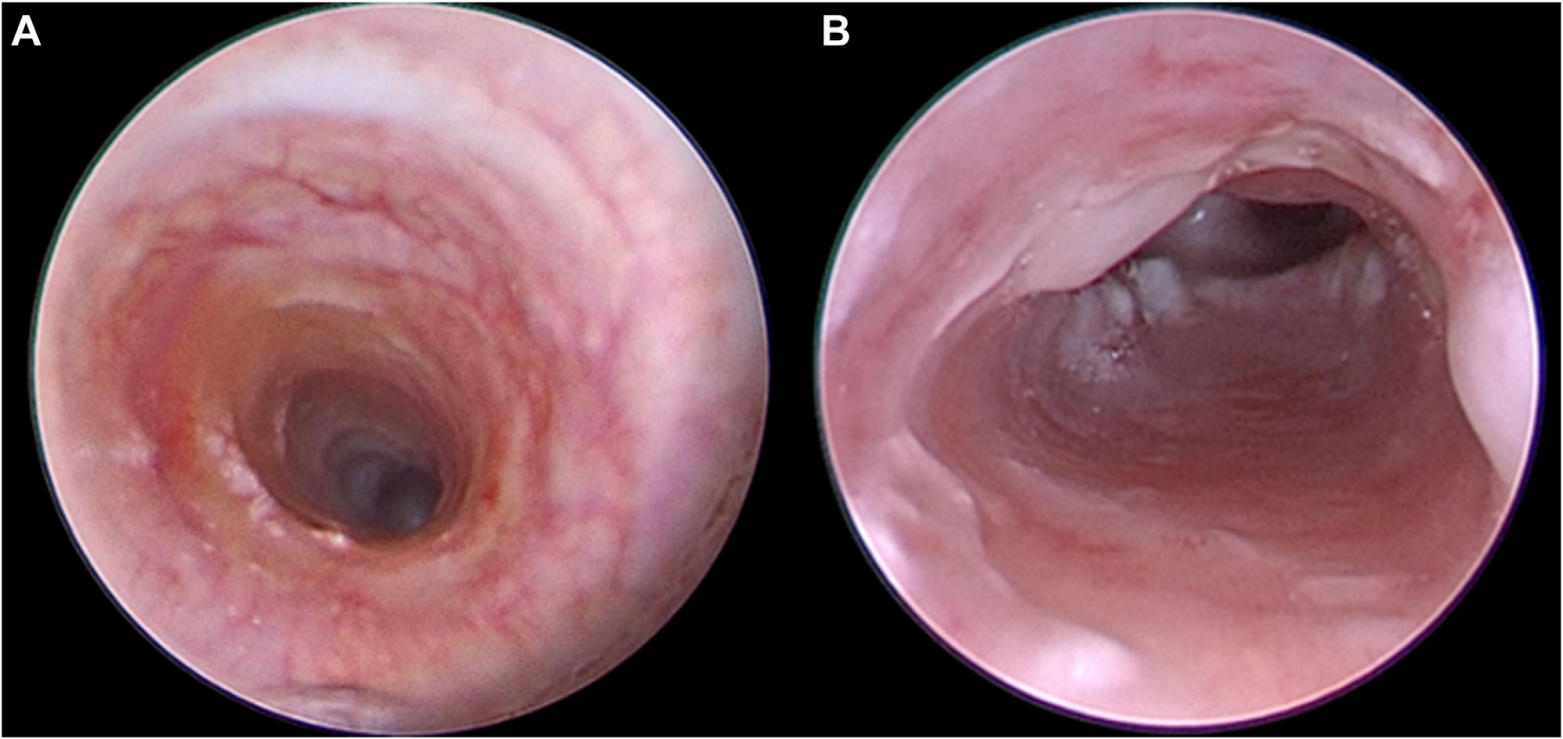
Perioperative bronchoscopy. (A) Preoperative long-segment complete tracheal rings. (B) Postoperative patent airway 2 weeks after tracheal reconstruction.

Two multidisciplinary meetings were held. First, imaging was reviewed at the multidisciplinary pediatric cardiology case discussion conference. Multiple options, including proceeding with CoA repair or catheter-based intervention, were explored. Because an appropriately sized ETT could not be passed, the patient’s airway was at predictably prohibitive risk intraoperatively and postoperatively. Given the significantly decreased size and extent of tracheal narrowing, the consensus was that the patient should undergo concomitant slide tracheoplasty and CoA repair. The next discussion was before the procedure. This meeting focused on technical aspects and postoperative care. Attendees included anesthesiologists, cardiologists, intensivists, operating room nurses, surgeons (pediatric surgeon, cardiac surgeon, and otolaryngologist), pharmacists, and perfusionists. Perioperative details, including specific equipment, ETT sizes, operative sequence, postoperative neck positioning, and use of inhaled ciprofloxacin/dexamethasone (Ciprodex), were reviewed.^3^

After the parents’ consent was obtained, the 2-week-old patient was brought to the operating room. A transnasal fiberoptic intubation was performed with a 3.5-mm uncuffed ETT, which was positioned just above the stenosis. Arterial and central venous access was obtained. Median sternotomy was performed. An anterior pericardial flap was harvested. Central arterial aortic cannulation and atrial venous cannulation were performed. The patent ductus arteriosus was ligated. The midtrachea was identified at the takeoff of the right innominate artery. After mobilization, the aortic arch appeared to be adequately sized. The recurrent laryngeal nerve was identified. Once cooled to 18 °C, an aortic cross-clamp was applied and cardioplegia administered with prompt arrest. Deep hypothermic circulatory arrest was initiated. The ascending aortic arterial cannula was then redirected into the innominate artery and antegrade cerebral perfusion started. The CoA segment was resected. An extended end-to-end anastomosis was performed with 7-0 Prolene running suture. The arterial cannula was then redirected into the ascending aorta and cardiopulmonary bypass resumed.

During rewarming, slide tracheoplasty was performed. Release maneuver of the inferior pulmonary ligaments was performed. The trachea was mobilized from above the innominate artery to the carina. Under flexible bronchoscopic guidance, the proximal extent of the stenosis was identified by placing a 30-gauge needle into the tracheal cartilage. Finding the distal extent was difficult because passage of the 2.8-mm scope was challenging. The stenotic segment midportion was identified. The transection was beveled across 2 tracheal rings.^6^ Vertical incisions were made along the anterior superior and posterior inferior segments. With use of 7-0 PDS, the trachea was anastomosed with a running suture. After suturing of the posterior wall, a 3.5-mm cuffless ETT was advanced across the anastomosis. The anterior anastomosis was then completed. A leak test was performed; there was a small air leak at the superior trachea. Sutures were placed to reinforce the anastomosis. Tisseal (Baxter) was applied to the trachea. A pericardial flap was placed between the innominate artery and trachea^7^ (Figure 3), with air leak resolution. Total cardiopulmonary bypass time was 246 minutes; cross-clamp time, 53 minutes; deep hypothermic circulatory arrest, 8 minutes; and antegrade cerebral perfusion, 41 minutes. The sternum was left open.

**Figure 3:**
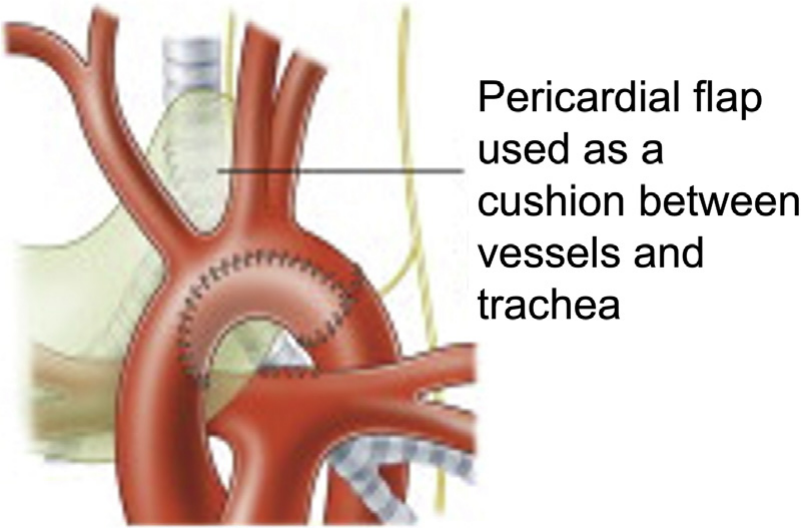
Pericardial flap buttressing tracheal repair. Reprinted from^7^ with permission from The Society of Thoracic Surgeons.

The patient had an uncomplicated postoperative course. On postoperative day (POD) 3, the sternum was closed. Bronchoscopy on POD 3 and POD 10 demonstrated bilateral vocal cord movement, intact anastomosis, and minimal granulation tissue (Figure 2B). Swallow testing on POD 14 was negative for aspiration. The patient tolerated oral feeding and was discharged home on POD 21.

## Comment

With recently evolving surgical techniques, practice variation still exists.^4^ However, many institutions prefer slide tracheoplasty for long-segment complete tracheal rings with simultaneous repair of intracardiac anomalies.^2^^,^^3^^,^^8^ Brink and colleagues^7^ described reinforcing the trachea with pericardium (Figure 3). Also, use of inhaled Ciprodex has decreased granuloma formation.^3^ With growing experience in pediatric concomitant tracheal repair and congenital cardiac operations, outcomes continue to improve.

This newborn case models solid practices for congenital cardiac centers. Foremost, every neonate with difficult intubation, regardless of respiratory symptoms, should have complete upper and lower airway evaluation before undergoing cardiac operation. This includes direct laryngoscopy, bronchoscopy, and cross-sectional imaging. If complete tracheal rings are identified, a multidisciplinary conference is necessary to determine whether concomitant airway and cardiac operations is advisable. Increasing experience in neonatal cardiac surgery and airway reconstruction continues to ensure that this is a feasible and preferred option.

In conclusion, the incidence of severe tracheal stenosis in asymptomatic neonates and congenital heart disease is unknown. Rarely, asymptomatic tracheal stenosis is discovered early through inability to intubate for a neonatal cardiac operation, such as CoA. With advancing surgical care, concomitant tracheal reconstruction and congenital cardiac operation continue to be safe and should be the preferred method in treating congenital cardiac lesions beyond the pulmonary artery sling.
